# Integrating mind and body: Investigating differential activation of nodes of the default mode network

**DOI:** 10.3233/RNN-231334

**Published:** 2023-12-08

**Authors:** Inbal Linchevski, Amber Maimon, Yulia Golland, Noa Zeharia, Amir Amedi, Nava Levit-Binnun

**Affiliations:** a Sagol Center for Brain and Mind, Baruch Ivcher School of Psychology, Reichman University, Herzliya, Israel; b The Baruch Ivcher Institute for Brain, Cognition and Technology, Baruch Ivcher School of Psychology, Reichman University, Herzliya, Israel; c The Ruth & Meir Rosental Brain Imaging (MRI) Center, Reichman University, Herzliya, Israel

**Keywords:** Progressive muscle relaxation, default mode network, DMN, self-referential processes, self-reference, body-mind

## Abstract

**Background:**

The default mode network (DMN) is a large-scale brain network tightly correlated with self and self-referential processing, activated by intrinsic tasks and deactivated by externally-directed tasks.

**Objective:**

In this study, we aim to investigate the novel approach of default mode activation during progressive muscle relaxation and examine whether differential activation patterns result from the movement of different body parts.

**Methods:**

We employed neuroimaging to investigate DMN activity during simple body movements, while performing progressive muscle relaxation. We focused on differentiating the neural response between facial movements and movements of other body parts.

**Results:**

Our results show that the movement of different body parts led to deactivation in several DMN nodes, namely the temporal poles, hippocampus, medial prefrontal cortex (mPFC), and posterior cingulate cortex. However, facial movement induced an inverted and selective positive BOLD pattern in some of these areas precisely. Moreover, areas in the temporal poles selective for face movement showed functional connectivity not only with the hippocampus and mPFC but also with the nucleus accumbens.

**Conclusions:**

Our findings suggest that both conceptual and embodied self-related processes, including body movements during progressive muscle relaxation, may be mapped onto shared brain networks. This could enhance our understanding of how practices like PMR influence DMN activity and potentially offer insights to inform therapeutic strategies that rely on mindful body movements.

## 1 Introduction

A most profound problem in intellectual history was and remains the problem of delineating the relationship between the mental self and the physical body (Northoff & Bermpohl, 2004). Neuroscience has recently begun to shed light on the mechanisms in the brain that underlie self-reference, with the spotlight shining specifically on what is known as the default mode network (DMN) (Northoff, 2011; Northoff & Bermpohl, 2004). The DMN is a large-scale brain network containing the medial prefrontal cortex, the posterior cingulate cortex, the temporoparietal junction, and the lateral and medial temporal lobes (Andrews-Hanna, Reidler, Huang, et al., 2010; Andrews-Hanna, Reidler, Sepulcre, et al., 2010; Raichle, 2015). On the one hand, the DMN is consistently deactivated by cognitively demanding goal-directed tasks, such as mental calculation (Shulman et al., 1997) and sensory processing tasks (Mazoyer et al., 2001). On the other hand, the DMN shows positive activity when people engage in tasks requiring internal mentation, such as self-reflection (Axelrod et al., 2017; Buckner & Carroll, 2007; Buckner & DiNicola, 2019; Goldberg et al., 2006), relative to a baseline rest condition. As such, the DMN has been postulated as a primary neural circuit involved in self-related processing (Buckner et al., 2008; Davey et al., 2016; Gusnard et al., 2001; Northoff & Bermpohl, 2004).

A central component of self-related processing is the information associated with one’s body. According to William James, the philosopher and psychologist, “Our own bodily position, attitude, condition, is one of the things of which some awareness, however inattentive, invariably accompanies the knowledge of whatever else we know” (McDermott, 2013). As such, body-related processes, such as somatosensation and motion, as well as more complex phenomena like body ownership and agency (the sense that I am the author of my body’s actions), have been generally tied to different aspects of the core self (Buckner et al., 2008; S. Gallagher, 2007; Tsakiris, 2017). Body characteristics, such as body weight, body shape, and fitness level, are incorporated into subjects’ self-concept and identity (Spencer-Oatey, 2007; Webster & Tiggemann, 2003), and self-consciousness has been postulated to rely on the multisensory processing of bodily signals (Blanke et al., 2015; Spencer-Oatey, 2007; Tsakiris, 2017; Webster & Tiggemann, 2003). Techniques like progressive muscle relaxation (PMR), which involve intentional sequential movements of body parts, serve as meditative tools for enhanced self-awareness. PMR involves tensing and then releasing different muscle groups, which promotes a heightened sense of one’s physical presence and can foster a mental state of relaxation and tranquility (Dolbier & Rush, 2012; Masmouei et al., 2019). In practices such as PMR, body characteristics and movements, that can be associated with personal identity and self-concept, integrate with the multisensory processing of bodily signals to contribute to self-consciousness. The question can be raised, how such body-related processes should affect the DMN. Such processes could be regarded as self-referential and would be expected to *activate* DMN regions (i.e., positive BOLD relative to baseline). For example, the movement of body parts like the face and trunk may be of particular relevance to self-related processes (Blanke et al., 2015; Blanke & Metzinger, 2009; Kim et al., 2014; McIntosh, 1996; Petkova & Ehrsson, 2008; Sugiura, 2015; Sui & Humphreys, 2017).

Facial movements are fundamental to personal identity and emotional interpretation, serving as a distinguishing factor in cognitive and social development, and establishing the self-other distinction (Sugiura, 2015; Sui & Humphreys, 2017). The facial feedback hypothesis further emphasizes their importance, suggesting that our emotional experiences are influenced by facial muscle feedback, impacting emotional state and potentially mitigating depression symptoms (Buck, 1980; Kim et al., 2014; McIntosh, 1996). Thus, facial movements are not only crucial to self-recognition but also play a significant role in interpreting our mental state and emotional reactions, reinforcing their centrality in the perception of the self (McIntosh, 1996; Sugiura, 2015; Sui & Humphreys, 2017). Following this, we hypothesize that the movement of different areas during a progressive muscle scan technique may activate the DMN, and possibly show differential effects on various nodes (Andrews-Hanna, Reidler, Huang, et al., 2010; Buckner et al., 2008; Fan et al., 2017; Salomon et al., 2014). By investigating DMN activation and deactivation patterns, and employing resting-state functional connectivity to further probe specific DMN nodes activated by specific body parts, we aim to contribute a nuanced understanding of how practices like PMR could potentially influence DMN activity.

Gaining a clear understanding of the default mode network is of particular significance as the DMN is known to be involved in several neurological and psychiatric disorders, among them Alzheimer’s disease, autism, schizophrenia, and depression (Broyd et al., 2009; Whitfield-Gabrieli & Ford, 2012). This is particularly relevant to the present study exploring self referential cognition and self related processing as these are known to be abnormal in or otherwise associated with the previously mentioned conditions, and more. Interventions like PMR, which encourage mindful attention to body sensations and movements, have been explored as techniques employed in managing these disorders. By investigating DMN activity during body movements akin to PMR, our study may potentially offer insights to inform such therapeutic strategies further.

## 2 Material and methods

### 2.1 Subjects

In this study, we apply a new set of hypotheses and analysis approaches to data reported in (Zeharia et al., 2015). Notably, we formulated our hypothesis prior to performing any statistical testing in accordance with norms in the field (Kerr, 1998). In the main experiment, eleven healthy, right-handed subjects aged 25–35 years participated in a body movement experiment. Our study utilized a smaller sample size akin to earlier fMRI studies as we used the same subjects and data as in (Zeharia et al., 2015). In addition, data from thirty-three participants who did not participate in the main experiment were analyzed for resting-state functional connectivity (Zeharia et al., 2015). All experimental procedures were approved by the Hebrew University ethics committee, and written informed consent was obtained from all subjects. All methods were performed in accordance with the relevant guidelines and regulations.

### 2.2 Progressive muscle relaxation experiment

#### 2.2.1 Experimental design

During the PMR experiment, subjects (*N* = 11) were requested to lie with their eyes closed and blindfolded inside the fMRI scanner and move twenty body parts consecutively, in two different sequence orders. The first movement sequence followed the order from toes to tongue, while in the second movement sequence, the movement was reversed. Sequences were in line with the order of body parts in the homunculus, as described by (Penfield & Boldrey, 1937) see (Zeharia et al., 2012) for details. It included bilateral movements of the following body parts: toes (flexion/extension), feet flexion/extension), thighs (contraction), buttocks (contraction), stomach (contraction), upper arm (contraction), elbow (flexion/extension), wrist (flexion/extension), fist (contraction), little finger (flexion/extension), ring finger (flexion/extension), middle finger (flexion/extension), index finger (flexion/extension), thumb (flexion/extension), forehead (contraction), nose (contraction), eyelids (contraction), lips (contraction), jaw (flexion/extension), and tongue (a side to side movement with the mouth closed).

The subjects were instructed to execute the movement of each specific body part upon hearing an auditory cue, which was the spoken name of that body part. This was followed by three metronome beeps at 1 s intervals, which paced the movement. Consequently, each body part was moved three times within a period of 3 s, and the next body part was announced during the last second. The whole movement sequence lasted 60 s and was followed by a rest period of 12 s in which subjects lay without moving and with closed eyes. Eight cycles of movement and rest were performed at a stimulus frequency of 0.0138 Hz. The subjects were trained for 1 h before entering the scanner. Eight out of the eleven participants were able to complete both the toes-to-tongue and the tongue-to-toes sequences. The remainder of the participants performed only the toes-to-tongue sequence due to technical reasons. Since similar results were found when we repeated the analyses with only the subset of eight participants who completed both sequences, we report here on the full sample (*N* = 11).

#### 2.2.2 Functional MRI acquisition

The BOLD fMRI measurements were obtained in a whole-body 3T Magnetom Trio scanner with 12 channels (Siemens). The fMRI protocols were based on multislice gradient echoplanar imaging and a standard head coil. The functional data were collected under the following timing parameters: TR = 1.5 s; TE = 30 ms; FA = 70°; imaging matrix, 80X80; FOV, 24X24 cm (i.e., in-plane resolution of 3 mm). Twenty-six slices with slice thickness of 4.5 mm and no gaps were oriented in the axial position for complete coverage of the cortex.

#### 2.2.3 Three-dimensional recording and cortex reconstruction

Separate 3D recordings were used for co-registration. High-resolution 3D anatomical volumes were collected using T1-weighted images using a 3D turbo field echo T1-weighted sequence (equivalent to MP-RAGE). Typical parameters were as follows: FOV, 23 (right to left) X23 (ventral to dorsal) X17 (anterior to posterior) cm; foldover axis: right to left; data matrix: 160 X 160 X144; zero filled to 256 in all directions (1 mm isovoxel native data); TR/TE=9/6 ms; flip angle = 8°. Group results were superimposed on a 3D cortical reconstruction of a Talairach-normalized brain (Laitinen, 1989).

### 2.3 Data analysis

#### 2.3.1 Preprocessing

The first ten images (during the first baseline rest condition) were excluded from the analysis because of non-steady-state magnetization. Data were preprocessed using the Brain Voyager 21.4 software package (Brain Innovation; Maastricht, Holland). Functional MRI data preprocessing included head-motion correction. Slice scan time correction and high-pass filtering using temporal smoothing in the frequency domain removed drifts and improved the signal-to-noise ratio. A 4mm-FWHM kernel was used for smoothing the data. Functional and anatomical datasets for each subject were aligned and fitted to standardized Talairach space. In this process, the data were resampled at a 3X3X3 mm resolution using a trilinear interpolation method. Finally, similar to (Zeharia et al., 2015) the twenty body parts were divided into subgroups. In our case, this resulted in four groups: hands (9 body parts), legs (3 body parts), trunk (2 body parts), and face (6 body parts)(Zeharia et al., 2015).

#### 2.3.2 Whole brain GLM analysis

To address our first question regarding patterns of activation and deactivation of DMN regions associated with externally guided movement of different body parts, we contrasted the movement period (grouping all body parts) vs. the rest-condition baseline. To address our second question, regarding the particular role of specific body areas, we contrasted one group of body parts with the other three groups. For example, the face group was contrasted with the hands, legs, and trunk groups; or the trunk group was contrasted with the face, hands, and legs groups. To verify the significance of our results, we used two different types of multiple comparisons corrections, Monte Carlo (Forman et al., 1995) and FDR (Benjamini & Hochberg, 1995) corrections. For the Monte Carlo correction, we used an initial *p*-value of *p* < 0.005 uncorrected and 1000 iterations using a gray matter mask, the FDR results are reported in an FDR-corrected *q* < 0.05.

#### 2.3.3 Regions of interest (ROI) analysis

Based on the whole-brain analysis results, ROIs were defined for further investigation. These ROIs were defined as voxels that demonstrated significant BOLD activations for face vs. other body parts, trunk vs. other body parts, legs vs other body parts and hands vs other body parts outside of the classical homunculi. For the contrasts of trunk, legs and hands vs. other body parts, we did not find any significant activation outside the typical homunculi so no ROIs were defined. On the other hand, the face vs. other body parts showed three bilateral ROIs outside the classical homunculi that exhibited positive BOLD response. We then set out to determine whether the contrast between the face vs. other body parts produced a positive BOLD response due to increased face-movement activation or rather a decreased face-movement deactivation. For this, in each ROI, we inspected the sign (negative/positive) of the parameter estimator for each body part (vs. rest). Only ROIs in which the parameter estimator for the face was significantly positive later served as the seed for resting-state functional connectivity analysis.

#### 2.3.4 Resting-state experimental design

In order to test the functional connectivity patterns of the ROIs that were identified for positive face-movement (see previous section), we analyzed an independent dataset (*N* = 33) of spontaneous BOLD fluctuations (Biswal et al., 1995; Zeharia et al., 2015). This data was collected while the subjects lay supine in the scanner with their eyes closed and without any external stimulation or task.

#### 2.3.5 MRI acquisition and data pre-processing

The functional data were collected under the following timing parameters: TR = 3 s; TE = 30 ms; FA = 90°; imaging matrix = 64X64; FOV = 24X24 cm. To obtain full coverage of the subjects’ brains, 29–46 slices of 4 mm thickness were used. The number of whole-brain images in each functional scan varied and were all cropped to 180 images. The first two images of each scan were excluded from the analysis because of non-steady-state magnetization. The ventricles and white matter signals were sampled using a grow-region function embedded in the Brain Voyager from a seed in each individual brain. Using MATLAB, ventricles and white matter time courses were regressed out of the data, and the resulting time-course was filtered to a frequency band width of 0.1–0.01 Hz (in which typical spontaneous BOLD fluctuations occur). The data were 8 mm spatially-smoothed.

#### 2.3.6 Functional connectivity analysis

In order to generate a map of the DMN, resting-state functional connectivity analysis was performed from two a priori defined sphere ROIs in two nodes of the DMN, as previously described (Greicius et al., 2003). The size of the ROI spheres was 443 voxels. These ROIs are the posterior cingulate cortex (PCC; Talairach coordinates –12, –47, 32) and the ventral anterior cingulate cortex (vACC; 6, 39, –4).

**Fig. 1:**
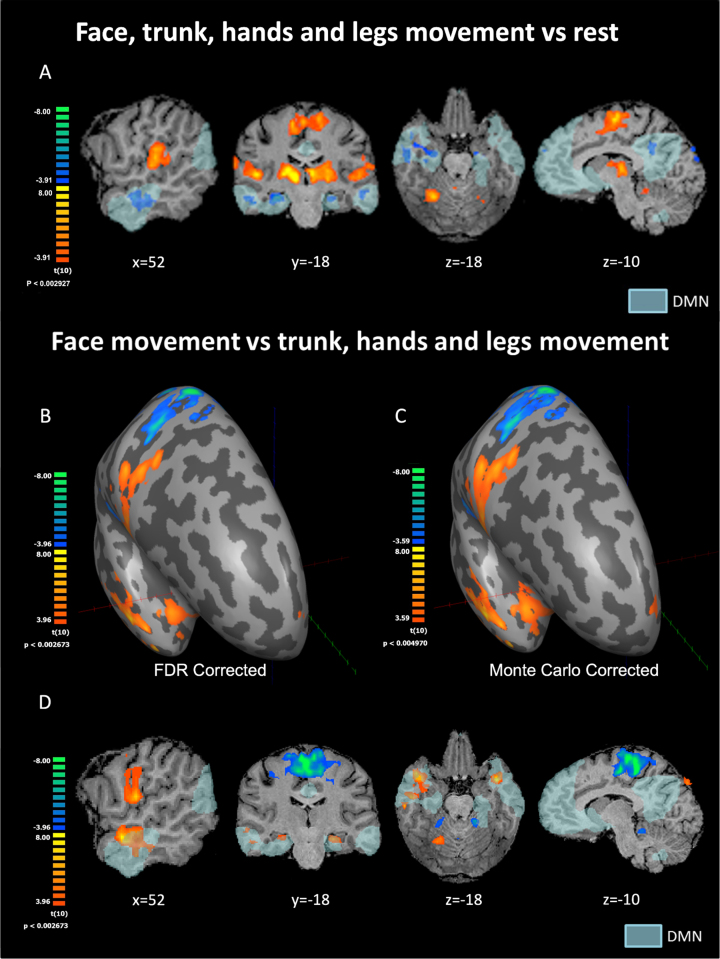
(A) Statistical parametric maps for movement of all body parts relative to rest condition following FDR correction. (B, C, D) Statistical parametric maps for face-movement relative to the movement of all other body parts. Results are presented on the inflated right hemisphere following FDR correction (B) and Monte Carlo correction (C), as well as on volume-based maps (D). The most intriguing activation pattern can be seen in B–D with strong face selectivity in the TP and Hip parts of the DMN. TP = temporal pole, Hip = hippocampus, PCC = posterior cingulate cortex, M1 = primary motor cortex, DMN = default mode network. DMN in light blue as derived from resting-state functional connectivity analysis.

**Fig. 2:**
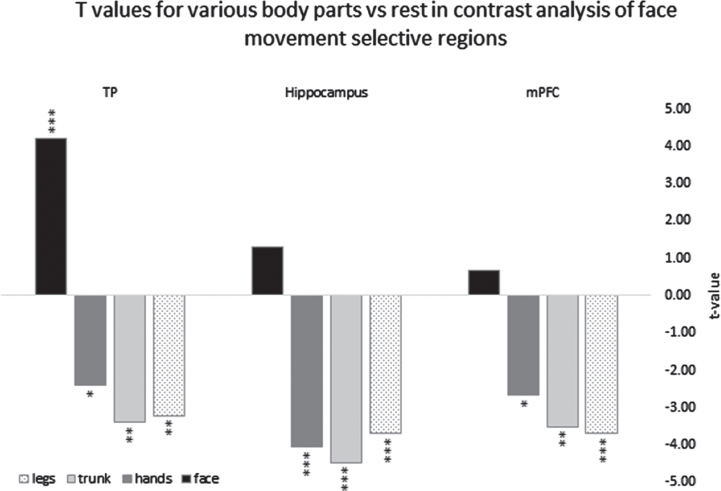
Parameter estimators for the contrasts of different body parts vs. rest derived from ROI GLM of the right hemisphere clusters in TP = temporal pole, Hippocampus, mPFC = medial prefrontal cortex, *=p < 0.05, **=p < 0.01, ***=p < 0.005.

We then used the two face-selective regions that were found in the body movement experiment (bilateral temporal poles, see Figs. 1B–D and 2) for the seed-based analysis. Time series from the resting-state scan for each subject were extracted for the ROIs in the right and left temporal poles by averaging the time series of all voxels in the ROI. The resulting time series were z-normalized and then used as seeds in a whole-brain linear correlation analysis. Linear correlation maps were created for each subject individually (single-subject analysis) for each ROI, using FDR multiple-comparisons correction of *q* < 0.05 (Biswal et al., 1995; Fox & Raichle, 2007; Striem-Amit et al., 2015). Then, a probabilistic map for all thirty-three subjects was computed at a minimal threshold of 60% overlap across the 33 individual subjects (Biswal et al., 1995; De Reus & Van Den Heuvel, 2013).

## 3 Results

### 3.1 Patterns of activation and deactivation collapsed across the movement of all body parts

We conducted a whole-brain random-effects GLM for the contrast between movement periods of all body parts vs. rest. Throughout the results section, t and p value reported are prior to applying the FDR correction. We found significant positive BOLD (see figure 1A) in the M1 homunculus and other parts of the sensory-motor system, in line with previous findings (Ashida et al., 2019; Penfield & Boldrey, 1937; Rowe & Siebner, 2012; Zeharia et al., 2015). Regarding DMN regions, clear and robust deactivation in the movement condition compared to rest was found in the temporal gyrus and temporal pole (significance of 4mm-FWHM-smoothed peak voxels in the right hemisphere: *t* = –7.08; *p* < 0.00005; Left hemisphere: *t* = –7.04; *p* < 0.00005), hippocampus (Right: *t* = –7.11; *p* < 0.00005; Left: *t* = –7.51; *p* < 0.00005), PCC (Right: *t* = –5.01; *p* < 0.001; Left: *t* = –5.41; *p* < 0.0005) and mPFC (Right: *t* = –5.88; *p* < 0.0005; Left: *t* = –5.20; *p* < 0.0005) (figure 1A). These areas show negative BOLD to simple bilateral body movements collapsed across all body parts. Other nodes of the DMN did not show positive or negative BOLD relative to baseline rest condition for this contrast.

### 3.2 Selective patterns of activation and deactivation for face-movement

A whole-brain random-effects GLM analysis for the contrast of specific body parts versus the other body parts (i.e., face movement vs. all other parts, trunk vs. all other parts, legs vs. all other parts, and hands vs. all other parts) was conducted. All four contrasts produced the expected significant activations within the sensory-motor system (e.g., M1 and Cerebellum) (Ashida et al., 2019; Penfield & Boldrey, 1937; Rowe & Siebner, 2012; Zeharia et al., 2015). Outside the sensory-motor system, significant clusters were identified only for the contrast analysis of the face vs. other body parts (using a threshold of FDR corrected *q* < 0.05). Findings related to the classical sensory-motor system were reported previously (Zeharia et al., 2012). Here, we report on novel findings related to clusters outside the sensory-motor system.

The contrast analysis of the face vs. other body parts revealed three new bilateral areas selectively activated for face movement in the temporal poles, the hippocampi, and the medial prefrontal cortex (Fig. 1B–D). Values for peak voxels in the temporal pole (significance of 4mm-FWHM-smoothed peak voxels in the right hemisphere: *t* = 7.0; *p* < 0.00005; Left hemisphere: *t* = 8.0; *p* < 0.00001), the hippocampus (Right: *t* = 5.3; *p* < 0.0005; Left: *t* = 5.9; *p* < 0.0001) and the medial prefrontal cortex (mPFC) (Right: *t* = 5.3; *p* < 0.0005; Left: *t* = 4.9; *p* < 0.001).

In order to understand the effect size, we compared values of peak voxels in these six clusters to the M1 homunculus (significance of 4mm-FWHM-smoothed peak voxels in the right hemisphere: *t* = 8.48; *p* < 0.00001; Left hemisphere: *t* = 10.13; *p* < 0.000001 –Fig. 1B–D). The highest t-value outside the M1-homunculus face area was in the temporal pole. Notably, we found negative BOLD peaks for the face vs. other body parts in the classical homunculi regions dedicated to other body parts (e.g., the feet). This finding is in accordance with (Zeharia et al., 2012), and served as a sanity check for our data and analyses. Results using the Monte Carlo correction (using an initial p-value of *p* < 0.005 uncorrected) are presented in Fig. 1C. The FDR results reported in an FDR-corrected *p*-value of *q* < 0.05 are presented in Figs. 1B and 1D.

Next, we tested whether the positive BOLD contrast in the significant face-selective clusters resulted from less deactivation to face movement or rather greater activation (relative to rest baseline) compared to the other body parts. For each of these clusters, we therefore conducted a ROI-GLM-fit to define the sign of the beta-estimators of each body part. Each non-face body part showed robust deactivation (negative BOLD relative to rest) in bilateral temporal pole regions, hippocampus, and mPFC ROIs (replicating the whole body GLM results). On the other hand, the facial movements showed significant activation (positive BOLD relative to rest) in the temporal poles and no significant difference from rest in the hippocampus and the mPFC (see figure 2).

All six clusters were found within the boundaries of the DMN. This was confirmed by an external localizer (see methods) and by verifying Talairach and MNI coordinates from the literature (Altmann et al., 2015; Shirer et al., 2012). The specific location of nodes was verified by the Talairach Client tool (Lancaster et al., 1997). Both types of correction for multiple comparisons (Monte Carlo and FDR) yielded similar results.

These results indicate that, in the temporal poles, the hippocampi, and the mPFC, the activation patterns for facial movement are distinctive from the activation patterns for movement of other body parts. A graphical presentation of the right hemisphere is depicted in figure 2.

### 3.3 Functional connectivity of areas selectively responsive to facial movement

**Fig. 3:**
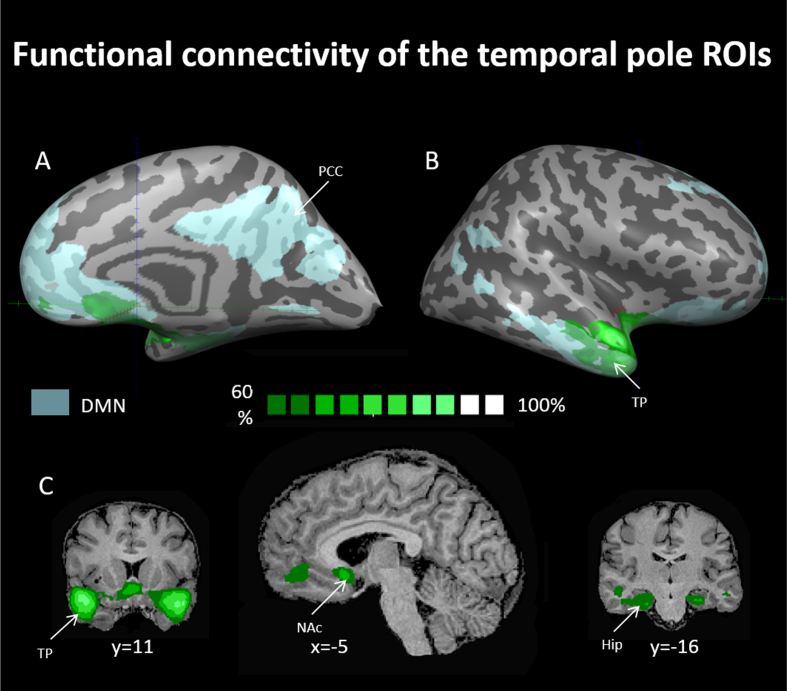
Probability maps for linear correlations of the face movement selective regions in the temporal poles using seed-based analysis. Maps are presented at a threshold of the majority of resting state subjects (60% probability and above –see dark green to bright green scale). ROIs for this analysis were chosen from the progressive muscle relaxation experiment and were used as seeds for computing linear correlations in the data of the resting-state experiment. TP = temporal pole, Hip = hippocampus, PCC = posterior cingulate cortex, NAcc = nucleus accumbens, DMN = default mode network. DMN in light blue as derived from rsFC analysis (see methods).

In order to further investigate these facial-movement-selective areas and their possible functions, we created two sphere ROIs for the right and left temporal pole (TP) regions. We then used these ROIs as seeds in whole-brain linear correlation maps in the data of subjects that participated in the resting-state experiment (see methods). A probabilistic map of regions exhibiting linear correlation to the TP-ROI seeds is presented in Fig. 3 (for a cutoff of 60% of all subjects). Within the DMN we observed functional connectivity of the TP ROI to the hippocampus and the mPFC, while outside the DMN, the TP ROIs were functionally connected to the nucleus accumbens. Identification of the mPFC (6, 39, –4) and nucleus accumbens (12, 7, –8) was done using coordinates taken from Greicius, 2003 and Cauda et al., 2011, respectively, and using the Talairach client tool (Lancaster et al., 1997). Identification of the hippocampus was done using the Talairach client tool as well.

## 4 Discussion

On the one hand, our findings are in line with prior research showing that voluntary body movements generally led to the deactivation of DMN regions. When collapsing across the movement of all body parts, we found consistent deactivation in several DMN regions, including the temporal poles and hippocampus, and to a lesser degree in the mPFC and PCC. Yet our findings indicate that not all DMN nodes responded to the movement of body parts with significant deactivation. For one, the tempo-parietal junction (TPJ) did not show a significant response (neither deactivation nor activation) to the movement of any of the body parts tested in this study. The TPJ has been implicated in embodiment, self-location, and imitation and thus may have a more complicated relationship with body parts (Blanke et al., 2005; Eddy, 2016).

As such, we suggest that different DMN nodes have differential roles in relation to body-related processes. This is in line with the growing understanding that there are sub-network specializations within the DMN (Andrews-Hanna, Reidler, Huang, et al., 2010; Axelrod et al., 2017; Salomon et al., 2014; Wen et al., 2020). Our resting state functional connectivity results, which include some DMN nodes (temporal poles, hippocampus, and mPFC) but not others (for example, not the PCC, precuneus, and TPJ), support this notion as well. We found that the temporal poles exhibited significant connectivity with the hippocampus and mPFC, but not other DMN nodes. With respect to body related processes such as PMR, the temporal poles may connect sensory information with personal feelings and memories, the hippocampus might be responsible for remembering and interpreting body-related experiences, while the mPFC likely oversees and evaluates the body’s states. Regarding why other DMN nodes like the PCC, precuneus, and TPJ were not implicated, we believe that the specific task of PMR could have engaged particular aspects of self-referential processing and sensory integration that these regions are more closely associated with, while other nodes may have different roles depending on the task or cognitive process, further strengthening the idea that different tasks may show differential effects on the various nodes of the DMN.

In addition, we showed that facial movements specifically lead to the selective activation of DMN regions. When analyzing different body parts’ movements separately, we found a positive BOLD activation pattern that was selective to simple repetitive facial movements. This selective activation to facial movement was observed in the temporal poles, hippocampus, and mPFC. In addition, these areas also showed functional connectivity with each other but not with other DMN nodes. Our findings show robust functional connectivity between the face-selective regions in the temporal poles and the nucleus accumbens, which is part of the brain’s reward system. We did not find a unique activation pattern for the movement of the trunk, hands, or legs. Our results set apart face movement from the movement of other body parts, including the trunk, in relation to self-referential processes.

We found that in the temporal poles, the positive BOLD response for facial movement vs. other body-part movements was due to a significant activation for face movement and significant deactivation for all other body parts. In the hippocampus and the mPFC, it resulted from significant deactivation for all body parts except the face, which was not different from rest. Notably, while a positive BOLD response to the contrast of face movement vs. other body parts’ movement was also previously found within the sensory-motor system (Zeharia et al., 2012), the patterns of activation were significantly different. Specifically, in M1, a significant activation for face movement and a significant deactivation for feet and hands movement was detected. However, in M1 similar findings were also found for the feet and the hands (i.e., the feet area in M1 exhibited activation for feet movement and deactivation for face and hands movement). These negative BOLD findings were interpreted by (Zeharia et al., 2012), to indicate a sharpening mechanism that serves to differentiate between body parts rather than to distinguish the face over the other body parts. For other parts of the sensory-motor system, such as the SMA and cerebellum, (Zeharia et al., 2012) did not detect negative BOLD responses to movement of the various body parts.

The differential reaction of the temporal poles, hippocampus, and medial prefrontal cortex to facial movement relative to other body parts’ movement is in line with growing work emphasizing the importance of the face for the concept and definition of the self (McIntosh, 1996; Sugiura, 2015; Sui & Humphreys, 2017). A range of tasks indicate that self-face processing is not identical to the processing of the faces of others. For example, people are significantly faster at recognizing self-face than other familiar faces (Sui & Humphreys, 2017), in making judgment decisions about their self-face (e.g., orientation of the face) (Sui & Han, 2007) and in forming associations to self-face (e.g., associating faces to shapes) (Sui & Humphreys, 2017). Indeed, accumulating evidence indicates that self-face recognition is considered a hallmark of cognitive and social development and separation between self and other (Sugiura, 2015).

Moreover, the specific role of facial movement in interpreting self mental states is exemplified in studies that support the facial feedback hypothesis, according to which one’s emotional experience is affected by feedback signals from facial muscles (Buck, 1980; McIntosh, 1996). In addition, manipulating facial expressions can result in changes in subjective emotional states (Kim et al., 2014; McIntosh, 1996). The role of facial muscle movement in emotional processing is also demonstrated in cases in which temporary facial muscle paralysis (e.g., by Botox injections) can alter the ability to imitate facial expressions, affect the emotional processing of viewed emotional faces, and has even been suggested to have a mitigating effect on depression symptoms (Kim et al., 2014). All these suggest that the face is central to how one identifies oneself and interprets one’s mental state and emotional reactions, thus being instrumental in assessing the state of the self.

Importantly, in our study, although all the regions that responded to facial movement are known as part of the DMN, they did not display the same level of response. Of all the nodes, the temporal pole showed the most robust activation to face movement. Not only did it evidence a clear positive activation pattern in response to facial movement and clear negative deactivation in response to other body parts’ movements, t-values for this contrast were almost as high as in the M1 homunculus (see figure 1B–D and results section). The temporal pole has been previously linked to facial movement and perception. Degeneration of the anterior temporal lobe is accompanied by reduced production and recognition of emotional facial expressions (Miller et al., 1997; Mychack et al., 2001; Olson et al., 2007). Previous studies have also demonstrated temporal pole activation for recognition of familiar versus unfamiliar faces (Sugiura et al., 2001; Tsukiura et al., 2003) and emotional versus neutral faces (Blair et al., 1999; Tsukiura et al., 2003). The temporal pole has a well-established role in various emotional and social behaviors, such as forming self-relevant semantic memories (Buckner & Carroll, 2007; Kondo et al., 2003; Olson et al., 2007), linking emotion and perception and theory of mind abilities (H. L. Gallagher et al., 2002; Jimura et al., 2010; Mitchell et al., 2006; Olson et al., 2007).

In addition to the processes we have examined, other mechanisms might contribute to the observed effects in our study. One such mechanism could be the generation of efference copies during self-generated movements. The brain is known to create these copies to predict and subsequently reduce the perception of sensory signals initiated by the self, thereby distinguishing self from others (Kilteni et al., 2020). This concept introduces an intriguing additional dimension to our understanding of self-related processes and their underlying neural mechanisms. Future research could further investigate the role of efference copies in modulating the functional connectivity observed in our study. This suggests that the complexity of self-related processes may extend beyond our current focus and opens exciting avenues for future exploration.

Overall, we suggest that our findings highlight the connection between body and mind and present an opportunity to study complex self-related topics, as well as mental and emotional processes, through body-based experimental designs. Our results suggest that body-related motor processes, modulate activity of nodes of the DMN. Our connectivity results suggest that facial movement may consequently affect also the nucleus accumbens. Together, these areas are involved in a wide range of processes, including the core self, self-referential activity, theory of mind, affective regulation, decision-making, mood, motivation, and feelings of pleasure and satisfaction (Buckner et al., 2008; Floresco, 2015; Goldberg et al., 2006; Northoff & Bermpohl, 2004; Olson et al., 2007). They are also involved in human vulnerabilities such as mood disorders, anxiety, and addictive behavior (Fan et al., 2017; Floresco, 2015; Jimura et al., 2010; Sheline et al., 2009). Our findings open the possibility that these processes can be accessed and fine-tuned not only through direct emotional and psychological interventions but also through various body-based interventions.

This study employed a procedure of PMR, which involves systematically tensing and relaxing different muscle groups. Indeed, mindfulness-based practices, such as PMR and body scan meditation, have been found to have beneficial mental and emotional effects (Jha et al., 2010; Piet & Hougaard, 2011; Rouleau et al., 2015). Mindfulness interventions have also been shown to reduce mind wandering (Deyo et al., 2009; Jain et al., 2007), which is associated with increased DMN activity (Andrews-Hanna, Reidler, Huang, et al., 2010; Buckner et al., 2008) and with unhappiness (Killingsworth & Gilbert, 2010). As the face seems to exhibit a differential activation pattern, it would be interesting to investigate the psychological and affective effects of practices that specifically focus on the face (e.g., mental scanning of the face or facial postures in yoga) relative to practices that focus on other body parts.

In addition, mindfulness practices like PMR have been linked to physical benefits (Gupta et al., 2016; Ismail & Elgzar, 2018), as supported by a myriad of scientific literature supporting the intricate relationship between mental states and physical health. The ability of the mind to shape our physical conditions is increasingly recognized. Specific examples of positive health impacts of mindfulness and related practices include (but are not limited to) better cancer treatment outcomes (Corn et al., 2020), improved inflammation levels (Gardi et al., 2022; Ng et al., 2020), and the delay of cognitive decline, potentially staving off conditions like Alzheimer’s disease (Quintana-Hernandez et al., 2016; Quintana-Hernández et al., 2023). This aligns with the notion that by positively influencing brain activity and mental states through mindfulness exercises, we can not only enhance quality of life but also potentially mitigate the physical manifestations of various diseases. Such approaches as the ones employed here, can help elucidate mechanisms underlying the beneficial effects (Cebolla et al., 2017; Complementary & Medicine (US), 2000) of various mind-body interventions.

Our study is not free from limitations, including the modest sample size and the use of Talairach space for fMRI data normalization. The sample size, although small, was governed by practical constraints inherent in conducting intricate fMRI studies and ensuring high-quality data. Nonetheless, we understand that it potentially limits the generalizability of our results. Furthermore, we elected to normalize our fMRI data to Talairach space. Future research could address these limitations by employing larger sample sizes and considering the use of MNI space for a more generalized representation.

## Data/code availability statement

No code was used outside the Brain Voyager 21.4 software package (Brain Innovation; Maastricht, Holland) besides using MATLAB.

Data is still being analyzed in other projects, and will subsequently be made available via Mendeley Data open repository
